# NCOA4 inhibits glioma progression by suppressing the Sonic Hedgehog pathway and its overexpression indicates a better glioma prognosis

**DOI:** 10.1007/s13258-025-01666-3

**Published:** 2025-08-12

**Authors:** Kaining Liu, Hu Wang, Tian Qiu, Guangxiu Wang, Anling Zhang, Zhifan Jia, Xiaoguang Tong

**Affiliations:** 1 Department of Neurosurgery, Huanhu Hospital Affiliated to Tianjin Medical University, 6 Jizhao Road, Tianjin, 300350 People’s Republic of China; 2 Department of Neurosurgery, Tianjin Medical University General Hospital, Tianjin Neurological Institute, Laboratory of Neuro-Oncology, Key Laboratory of Post-Trauma Neuro-Repair and Regeneration in Central Nervous System, Ministry of Education, Tianjin Key Laboratory of Injuries, Variations and Regeneration of Nervous System, 154 Anshan Road, Tianjin, 300052 People’s Republic of China

**Keywords:** NCOA4, Glioma progression, PTCH1, SHH pathway

## Abstract

**Background:**

Nuclear receptor coactivator 4 (NCOA4) is known to be involved in ferroptosis. However, its expression and function in gliomas are still unclear.

**Objective:**

To assess the expression of NCOA4 in gliomas and explore the mechanisms by which NCOA4 affects glioma progression.

**Methods:**

RNA-seq data for glioma patient tissues and normal brain tissues were obtained from The Cancer Genome Atlas and the Genotype Tissue Expression project. NCOA4 expression was assessed by Western blotting (WB) and immunohistochemistry (IHC). Overexpression and knockdown of NCOA4 were induced in glioma cell lines via transduction of recombinant adenovirus encoding NCOA4 and NCOA4 siRNA, respectively. Cell Counting Kit-8 (CCK-8), Transwell and flow cytometry assays were performed to assess cell proliferation, invasion and apoptosis.

**Results:**

WB and IHC revealed that NCOA4 was markedly downregulated in glioma cell lines and human specimens compared to controls, and high NCOA4 expression was associated with a better glioma prognosis. NCOA4 overexpression inhibited glioma cell growth and invasion and induced apoptosis, whereas NCOA4 knockdown promoted glioma cell growth. PTCH1 was predicted to interact with NCOA4 via bioinformatics analysis. NCOA4 overexpression increased the expression of PTCH1 and suppressed the expression of SMO, Bcl-2 and the nuclear translocation of Gli1, indicating that NCOA4 suppresses the SHH pathway. PTCH1 knockdown reversed the inhibitory effects of NCOA4 on the malignant behaviours of glioma cells.

**Conclusions:**

These results suggest that NCOA4 is downregulated in gliomas and that its overexpression predicts better overall survival in glioma patients. Mechanistically, NCOA4 overexpression inhibits the progression of glioma by suppressing the SHH pathway.

## Introduction

Glioma is the most common primary brain tumour and presents a formidable clinical challenge (Wang et al. 2023). Although the diagnosis and treatment of gliomas have greatly improved, the prognosis is still poor, and the median survival of patients with glioblastoma after diagnosis is only 12–15 months (Loui et al. 2021; Dolecek et al. 2012). Therefore, the major focus of glioma research is to explore the pathogenesis of gliomas and provide new approaches for clinical treatment; however, the molecular pathogenesis of glioma remains inadequately understood.

NCOA4 was initially identified in papillary thyroid carcinoma and is also referred to as androgen receptor-associated protein 70 (ARA70). Subsequent studies have revealed that NCOA4 acts as a coactivator for a variety of nuclear receptors, such as steroid hormones, thyroid hormones, aryl hydrocarbons and vitamins D and A (Kollara and Brown 2012). NCOA4 has also been shown to play a role in cellular ferroptosis and to exert tumour-suppressive effects in cancers. NCOA4 mRNA expression is decreased in both breast cancer and prostate cancer (Ligr et al. 2010). NCOA4 overexpression suppresses the proliferation of prostate cancer cell lines and MCF-7 breast cancer cells (Xinyu et al. 2011; Mestayer et al. 2003). NCOA4 can bind to ferritin and transfer it to the autophagosome and then to the lysosome for ferritin degradation and simultaneous iron release (Santana-Codina et al. 2021). Overexpression of NCOA4 has been reported to promote erastin-induced ferroptosis in pancreatic cancer cells (Hou et al. 2016). Reports of NCOA4 in gliomas have been limited thus far. HECW1 has been reported to suppress the proliferation of glioma cells by upregulating NCOA4, and TRIM7 can regulate the growth of glioma cells through NCOA4-mediated ferroptosis (Lin et al. 2023; Li et al. 2022). However, there is no relevant research indicating whether NCOA4 can regulate the progression of glioma through pathways other than ferroptosis.

Sonic Hedgehog (SHH) signalling is a complex pathway essential for regulating many developmental processes. PTCH1 is a transmembrane protein that functions as a Hedgehog (Hh) receptor in the SHH signalling pathway. In the absence of Hh, PTCH1 inhibits SMO, and the downstream transcription factor Gli1 is repressed sequentially. The SHH pathway is suppressed, and the expression of target genes of the SHH pathway, such as Bcl-2, is also downregulated (Wen-Zhong et al. 2013). Aberrant activation of the SHH signalling pathway has been closely connected to processes related to the initiation and progression of glioma, including proliferation, migration and invasion (Du et al. 2015; Li et al. 2016; Sigafoos et al. 2021).

In this study, we assessed the expression of NCOA4 in gliomas and the effect of NCOA4 on the proliferation of glioma cells and explored the mechanisms by which NCOA4 affects glioma progression.

## Methods and materials

### The expression of NCOA4 was analysed via the TCGA, GTEx and TIMER databases

RNA‑seq data and corresponding clinical information of glioma patients were obtained from The Cancer Genome Atlas (TCGA) (http://cancergenome.nih.gov/), and the mRNA-seq data of normal brain tissues were obtained from the Genotype–Tissue Expression (GTEx) project (https://www.gtexportal.org). Then, the mRNA data of TCGA and GTEx were merged and normalized via the R package “limma.” The dataset included information on a total of 432 glioma tissues and 1152 normal brain tissues. The expression levels of NCOA4 mRNA across cancers were investigated via TIMER2.0 (http://timer.comp-genomics.org), and the values of NCOA4 in different grade gliomas and normal brain tissues were compared in the training set and validation set via the R package limma. A *p* value < 0.05 was used as a threshold for significance.

### Investigation of the prognostic value of NCOA4 in gliomas

According to the median expression value of NCOA4, the tumour samples in the training and validation sets were divided into high- and low-expression groups. Kaplan–Meier (K–M) survival curves of glioma patients were generated with the R package “survival” (https://CRAN.R-project.org/package=survival).

### Reagents and consumables

Dulbecco's modified Eagle's medium (DMEM) (cat. no. 11320033), foetal bovine serum (FBS) (cat. no. A5256701) and 0.25% trypsin–EDTA (cat. no. 25200056) were purchased from Gibco Life Technologies (Geand Land, New York, USA).

Protease inhibitor (cat. no. P6730), RIPA (cat. no. R0010), dimethylsulfoxide (DMSO) (cat. no. D8371), antibiotics (penicillin‒streptomycin solution) (cat. no. P1400), SDS‒PAGE loading buffer (cat. no. S1051) and TRIzol (cat. no. 15596026) were obtained from Solarbio Life Science (Beijing, China). The Minute TM cytoplasmic and nuclear extraction kit (cat. no. SC-003) for separating cytoplasmic and nuclear proteins was obtained from Invent Biotechnologies (Beijing, China).

Antibodies against β-actin (cat. no. 20536-1-AP), PCNA (cat. no. 60097-1-Ig), caspase-3 (cat. no. 19677-1-AP) and Bcl-2 (cat. no. 26593-1-AP) were obtained from Proteintech (Wuhan, China). Antibodies against NCOA4 (cat. no. ab86707), PTCH1 (cat. no. ab53715) and SMO (cat. no. ab236465) were obtained from Abcam (Cambridge, UK). Antibodies against histone H3 (cat. no. 4499S) and MMP2 (cat. no. 4022S) were obtained from Cell Signaling Technology (Boston, USA).

The immunohistochemistry (IHC) kit (cat. no. PV-9005) was obtained from ZSGB-Bio Ltd. (Beijing, China). PVDF membranes (cat. no. IPVH00010) were obtained from Millipore (Bedford, MA, USA). Cell Counting Kit-8 (CCK-8) (cat. no. CK04) was purchased from Dojindo Molecular Technologies, Inc. (Kumamoto, Japan). Matrigel (cat. no. 356234) was obtained from Corning (New York, USA). Transwell inserts (8 µm pore size) (cat. no. CP012036) were obtained from Jet Bio-Filtration (Guangzhou, China).

The short interfering RNAs (siRNAs) were purchased from Aibosi Biotechnology (Shanghai, China). The recombinant adenovirus NCOA4 (Adv-NCOA4) was purchased from Jikai Biotechnology (Shanghai, China).

Tissue chips of glioma specimens were obtained from Weiao Biotechnology (Shanghai, China) Art. No. ZL-BraGsur1801) and embedded in paraffin; they included samples of 180 cases: 5 cases of WHO grade I glioma, 74 cases of WHO grade II glioma, 26 cases of WHO grade III glioma, and 75 cases of WHO grade IV glioma.

### Glioma cell lines and cell culture

The glioblastoma (GBM) cell lines A172, U87, 23N, TJ905, T98G, LN18, LN229, B2-17, U251 and SNB19 were preserved by the Neuro-Oncology Laboratory, Tianjin Institute of Neurology. Human astrocytes (ASs) were primary human cells purchased from Pricella (Wuhan, China) (cat. no. CP-H122). All the cells were authenticated through short tandem repeat profiling and subjected to mycoplasma testing. All the cell lines were cultured with DMEM containing 10% FBS (foetal bovine serum) in 37 °C, 5% CO2 incubator and subcultured every 2–3 days.

### Glioma specimens

Six glioma samples (grade I: 2 samples, grade II: 1 sample, grade III: 1 sample, and grade IV: 2 samples) and paired adjacent nontumorous brain tissues were collected and snap-frozen in liquid N2 for subsequent analysis; the glioma tissues and adjacent tissues were obtained from the Department of Neurosurgery, Tianjin Huanhu Hospital, from 2024 August ~ 2024 December from patients with gliomas who had undergone necessary surgical treatment. The age range of the patients was 46–63 years. Each glioma tissue sample and part of each nontumorous tissue sample were ground into powder in liquid nitrogen; 500 µl of RIPA buffer was added, ultrasonic decomposition was performed for 2 min, the mixture was incubated on ice for 20 min, the mixture was centrifuged at 12,000 × g for 15 min, the supernatant was collected, quarter 5 × SDS‒PAGE loading buffer was added, the mixture was boiled in a water bath for 5 min, and the mixture was stored at − 80 °C for WB. Some nontumorous tissues were preserved in formalin at 4 °C for IHC. This study was carried out in accordance with the principles of the Ethics Committee of Tianjin Huanhu Hospital.

### Immunohistochemistry (IHC)

IHC was performed according to a previously published study (Yubing Hao et al. 2023). The tissue chip and nontumorous brain tissues were incubated with the appropriate primary antibody (NCOA4, 1:50 dilution) in a 4 °C wet box overnight, incubated with secondary antibody (ZSGB-Bio Ltd., cat. no. PV-9005) for 20 min at 37 °C, and then subjected to DAB and haematoxylin staining, dehydration and sealing.

### Cell transfection and protein detection

When the density of cultured cells reached 70–80%, siRNA-NCOA4 and siRNA-PTCH1 (Table 1) were transfected into U251, TJ905, 23N and U87 cells via Lipofectamine 3000, and Adv-NCOA4 was transduced into U251 and TJ905 cells using ADV helper reagent (ADV-HR) according to the manufacturer’s instructions. Forty-eight hours after transfection, proteins were extracted using RIPA lysis buffer (PMSF, 1:100). Protein expression was detected by WB. Equal amounts of protein were separated on 10% SDS–PAGE gels.

**Table 1:** Sequences of siRNAs

ID	Sequence (5′‑3′ )
si‑NCOA4 #1	Sense: GAGAAGUGGUUAUAUCGAACU Antisense: UUCGAUAUAACCACUUCUCUU
si‑NCOA4 #2	Sense: GCUCAUGCUAGUUCAGCAAAU Antisense: UUGCUGAACUAGCAUGAGCCA
si‑NCOA4 #3	Sense: CAAUUGUCUUACUCAUCAACU Antisense: UUGAUGAGUAAGACAAUUGAA
si‑NC	Sense: UUCUCCGAACGUGUCACGUTT Antisense: ACGUGACACGUUCGGAGAATT
si‑PTCH1 #1	Sense: GCACUUACUUUACGACCUACA Antisense: UAGGUCGUAAAGUAAGUGCUG
si‑PTCH1 #2	Sense: GGAUCUGAGUUCGACUUCAUU Antisense: UGAAGUCGAACUCAGAUCCCG
si‑PTCH1 #3	Sense: GGUUACAUGGAUCAGAUAAUA Antisense: UUAUCUGAUCCAUGUAACCUG
si‑NC	Sense: UUCUCCGAACGUGUCACGUTT Antisense: ACGUGACACGUUCGGAGAATT

### Proliferation analysis

For the CCK-8 assay, 3 × 10^3^ glioma cells were inoculated into 96-well plates. After 2 h of incubation at 37 °C, the cell viability was assessed at 450 nm. The proliferation of the cells was assessed at 0, 24, 48, 72 and 96 h.

### Glioma cell invasion assay

The cells subjected to the indicated treatments were seeded in the upper chambers of 24-well plates at 2 × 10^4^ cells/well in serum-free medium; the chambers were precoated with Matrigel. Complete medium was added to the lower chamber. After 48 h of incubation, the cells were fixed with formaldehyde and stained with crystal violet; the number of invading cells in each chamber was determined by counting the cells in three fields under an inverted light microscope.

### Glioma cell apoptosis assay

The cells were harvested and suspended in 100 µL of binding buffer at a density of 1 × 10^5^ cells/mL. The cell suspension was then mixed with Annexin V-FITC conjugate and propidium iodide (PI) solution and incubated for 15 min at room temperature in the dark. Apoptotic cells were detected using a BD FACSCaliber flow cytometer and analysed via FlowJo 10.8 (Becton, Dickinson and Company).

#### Protein–protein interaction network (PPI) and functional enrichment analysis

The PPI network of NCOA4 was predicted via STRING (https://string-db.org/). A correlation coefficient of > 0.6 and a p value below 0.01 were considered to indicate significant coexpression. Gene Ontology (GO) and Kyoto Encyclopedia of Genes and Genomes (KEGG) analyses were subsequently performed via the R package “clusterProfiler” to investigate the potential functions and signalling pathways involved.

#### Statistical analysis

Statistical analysis was carried out via SPSS Statistics 25.0 (SPSS, Chicago, IL, USA). The differences between groups with normally distributed data (displayed as the mean ± standard deviation) were assessed via the two‑tailed unpaired Student's t‑test. The differences between groups with enumeration data were assessed via the χ2 test. *P < 0.05 was considered to indicate statistical significance. The graphs were generated with GraphPad Prism 6.0 (GraphPad Software, San Diego, California, USA).

## Results

### Downregulation of NCOA4 expression in gliomas

**Fig. 1:**
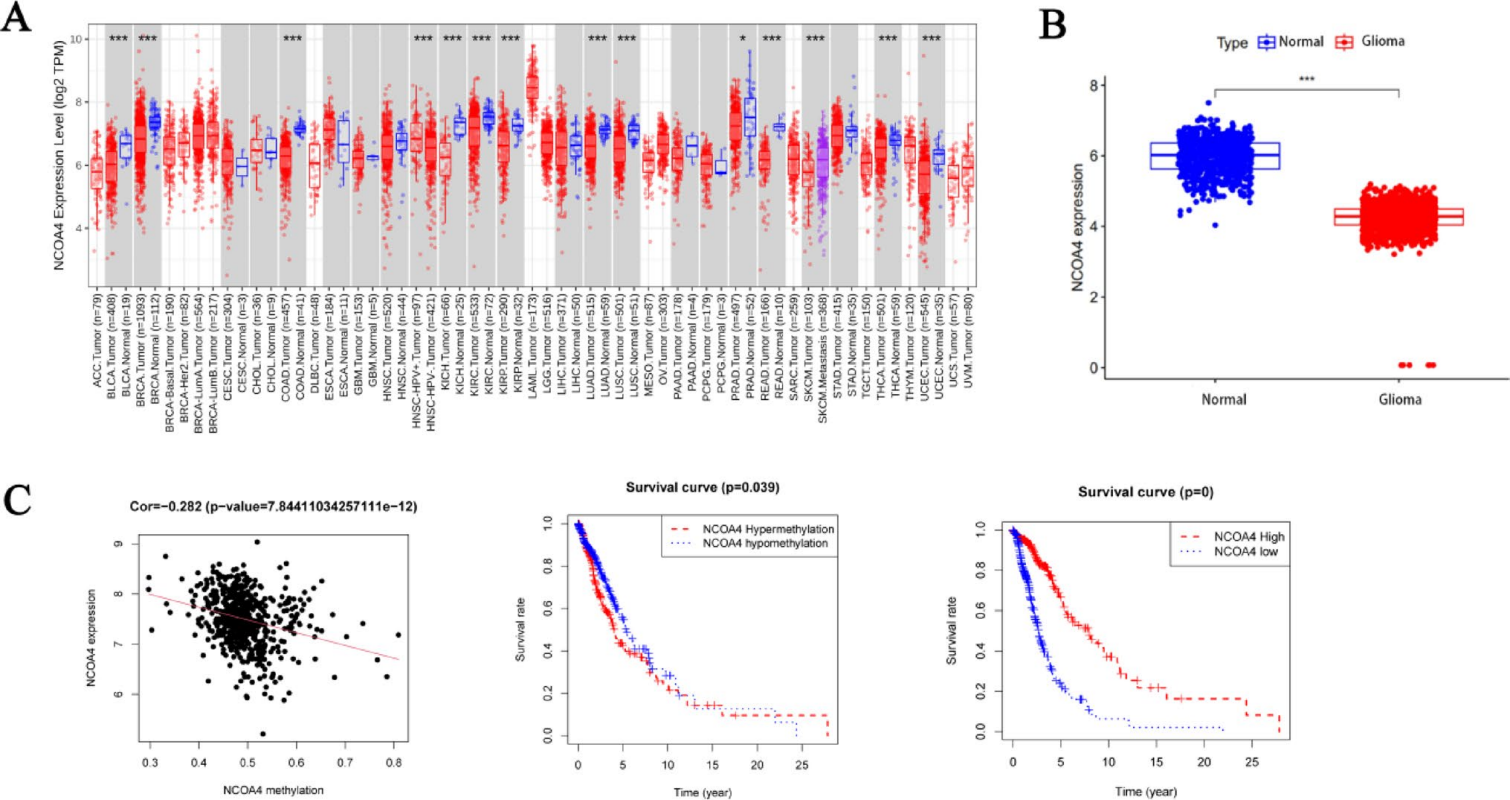
NCOA4 downregulation is observed in gliomas and indicates a poor prognosis. **A** NCOA4 is downregulated in various tumour tissues; blue indicates normal tissues, and red indicates tumour tissues. **B** NCOA4 in glioma tissues and normal tissues in the Cancer Genome Atlas (TCGA) and the Genotype–Tissue Expression (GTEx) project. **C** Kaplan‒Meier analysis of the overall survival (OS) of glioma patients with high/low NCOA4 expression and DNA methylation from the TCGA database

Pancancer analysis revealed that the NCOA4 expression level was lower in most solid cancer tissues than in normal tissues (Fig. 1A). The NCOA4 expression level was significantly lower in glioma tissues than in normal brain tissues in the training set (Fig. 1B). To preliminarily explore the mechanism underlying aberrant NCOA4 expression in glioma, we investigated the relationship between NCOA4 expression and DNA methylation; an obvious negative correlation (r =  − 0.282, *P* < 0.01) between NCOA4 expression and NCOA4 DNA methylation was observed (Fig. 1C). Moreover, analyses of Kaplan–Meier curves revealed glioma patients with high expression and low methylation of NCOA4 had better overall survival (OS) than those with low expression and high methylation of NCOA4 (*P* < 0.01) (Fig. 1C).

**Fig. 2:**
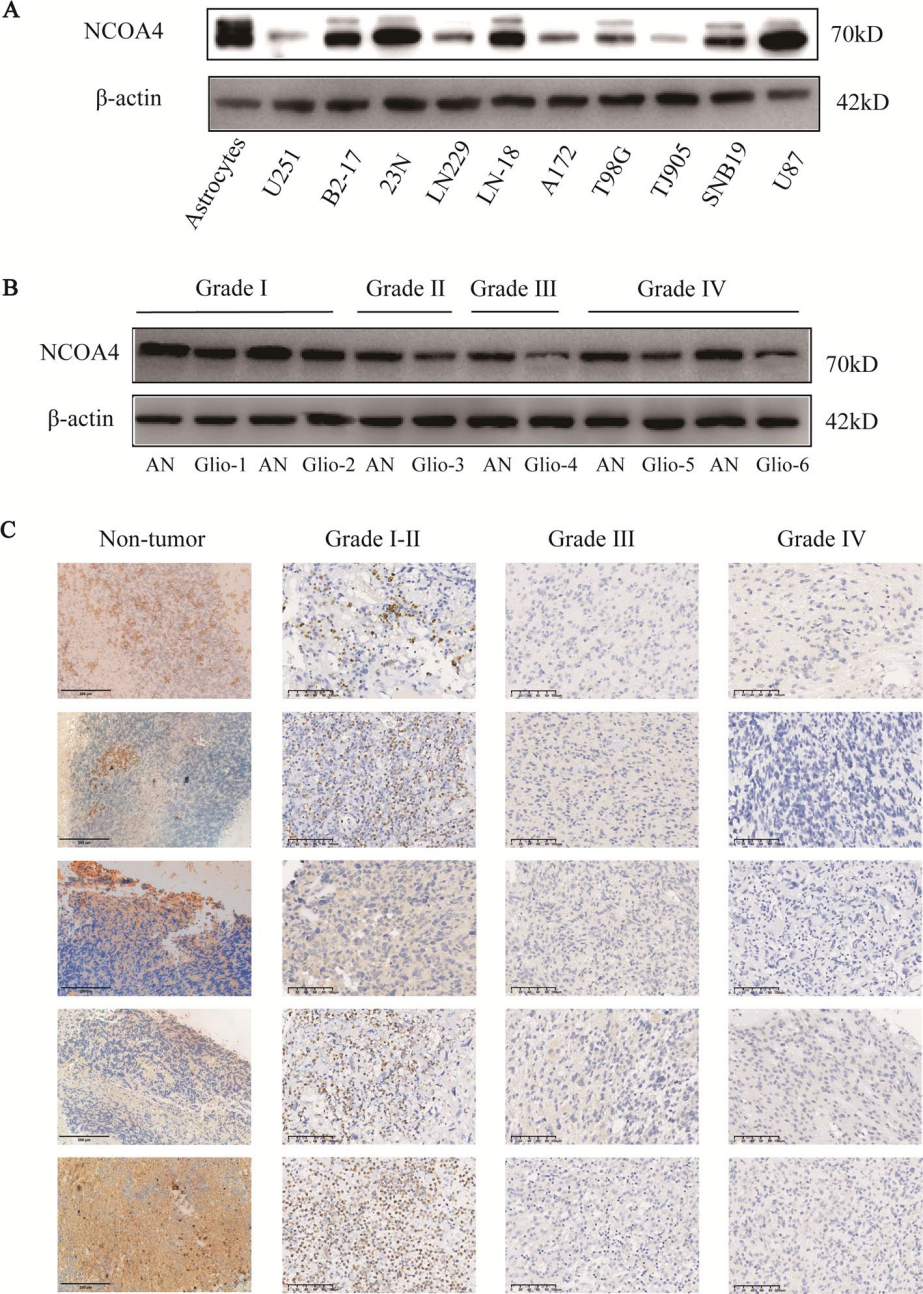
NCOA4 expression is downregulated in glioma cell lines and specimens. **A** The protein level of NCOA4 was analysed in glioma cell lines (A172, U87, 23N, TJ905, T98G, LN18, LN229, B2-17, U251 and SNB19) and astrocytes. **B** The protein expression of NCOA4 was analysed in glioma tissues and adjacent nontumorous (AN) brain tissues. **C** Expression of NCOA4 in glioma tissues from the tissue microarray and nontumorous tissues (× 200)

We further assessed the expression of NCOA4 in glioma cell lines and specimens by WB and IHC. NCOA4 expression was downregulated in most glioma cell lines compared with astrocytes; it was significantly decreased in LN229, TJ905, A172, T98G, U251 and SNB19 cell lines and slightly decreased in 23N, U87, LN18 and B2-17 cell lines (Fig. 2A). We assessed the expression of NCOA4 in 6 glioma samples and adjacent nontumorous (AN) brain tissues by WB. NCOA4 expression was downregulated in glioma samples compared with AN tissues and was negatively correlated with tumour grade (Fig. 2B). On the basis of the IHC staining data of NCOA4 in the tissue chip and nontumorous tissues, nontumorous tissues and low-grade gliomas exhibited intense staining, whereas NCOA4 was not detected in high-grade gliomas (Fig. 2C), which was consistent with the WB results.

We further analysed the correlation of the NCOA4 expression level of glioma samples from the tissue chip with tumour grade and patient OS, sex and age. The NCOA4 expression level was negatively correlated with the grade of glioma, and patients with positive NCOA4 staining had longer survival than those with negative NCOA4 staining. However, NCOA4 expression was not correlated with sex or age (Table 2).

**Table 2:** Correlations between NCOA4 expression and clinicopathological features

Clinicopathological features	Number of cases	NCOA4 expression		*P* value
		Positive	Negative	
All patients	180	79	101	
*Age (years)*				0.097
≤ 60	130	62	68	
> 60	50	17	33	
*Sex*				0.645
Male	115	49	66	
Female	65	30	35	
Overall survival (months)		22.08 ± 12.56	15.85 ± 7.15	0.001
*WHO grade*				0.007
I ~ II	79	42	37	
III	25	13	12	
IV	76	24	52	

### NCOA4 suppresses glioma progression

**Fig. 3:**
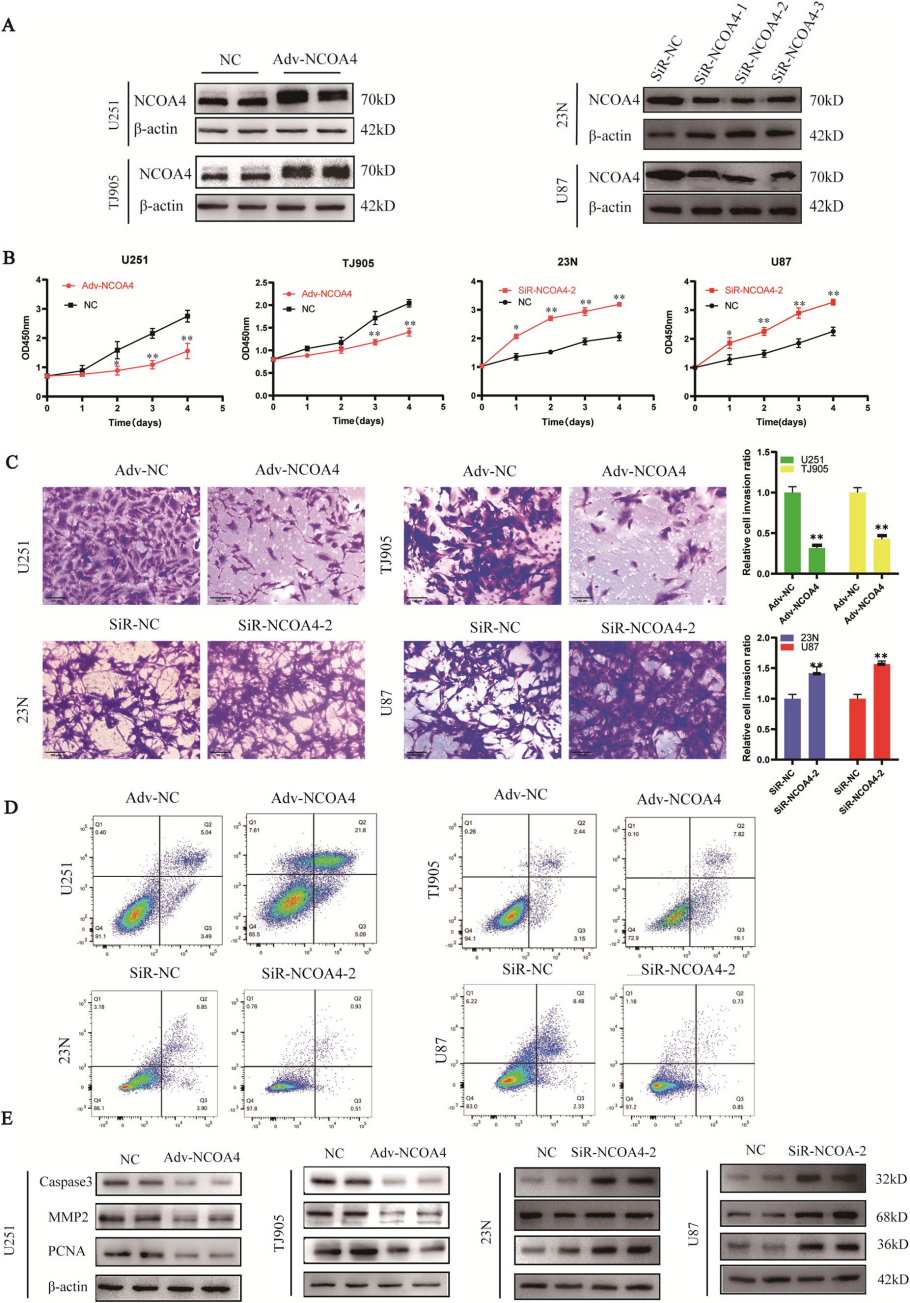
NCOA4 suppresses the growth of GBM cells. **A** NCOA4 knockdown and overexpression in glioma cell lines. **B** CCK-8 assay after NCOA4 knockdown and overexpression in glioma cell lines. **C** Invasion was detected via a Transwell assay (× 200). **D** Cell apoptosis was assessed via flow cytometry (*, *P* < 0.05; **, *P* < 0.01 compared with the control group). **E** Expression of MMP2, PCNA and cleaved caspase-3 in glioma cell lines transduced with Adv-NCOA4 and siR- NCOA4

To evaluate the effect of NCOA4 on the growth of glioma cells, Adv-NCOA4 was used to overexpress NCOA4 in U251 and TJ905 cells, and siRNA-NCOA4 was used to knockdown NCOA4 in 23N and U87 cells (Fig. 3A). The successful transduction of Adv-NCOA4 and siRNA-NCOA4 were detected by WB.

The CCK-8 assay results revealed that NCOA4 overexpression suppressed the proliferation of U251 and TJ905 cells. There was a significant difference in proliferative activity between the NCOA4-overexpressing group and the control group beginning on the third day (*P* < 0.05); conversely, NCOA4 knockdown promoted the proliferation of 23N and U87 cells. A significant difference in proliferative activity between the NCOA4 knockdown group and the control group was observed on the second day (*P* < 0.05) (Fig. 3B).

The number of invading cells was decreased when NCOA4 was overexpressed in U251 and TJ905 cells, and the number of invading cells was increased when NCOA4 was knocked down in 23N and U87 cells (Fig. 3C).

In addition, the cell apoptosis index was increased in NCOA4-overexpressing U251 and TJ905 cells compared with control cells, indicating that NCOA4 overexpression induces apoptosis. The apoptosis indices of 23N and U87 cells transfected with siR-NCOA4 were decreased, and NCOA4 knockdown suppressed glioma cell apoptosis (Fig. 3D).

Compared with those in the control groups, the expression levels of PCNA, MMP2 and Caspase3 were lower in U251 and TJ905 cells transduced with Adv-NCOA4, and the expression levels of PCNA, MMP2 and Caspase3 were greater in 23N and U87 cells transfected with siRNA-NCOA4 (Fig. 3E). These results were consistent with the effects of NCOA4 on cell proliferation, invasion, and apoptosis.

NCOA4 overexpression inhibited tumour cell proliferation and invasion and induced glioma cell apoptosis, whereas NCOA4 knockdown promoted glioma cell growth and invasion and suppressed apoptosis. These results suggest that NCOA4 suppresses glioma progression.

### PPI network construction and functional analysis

**Fig. 4:**
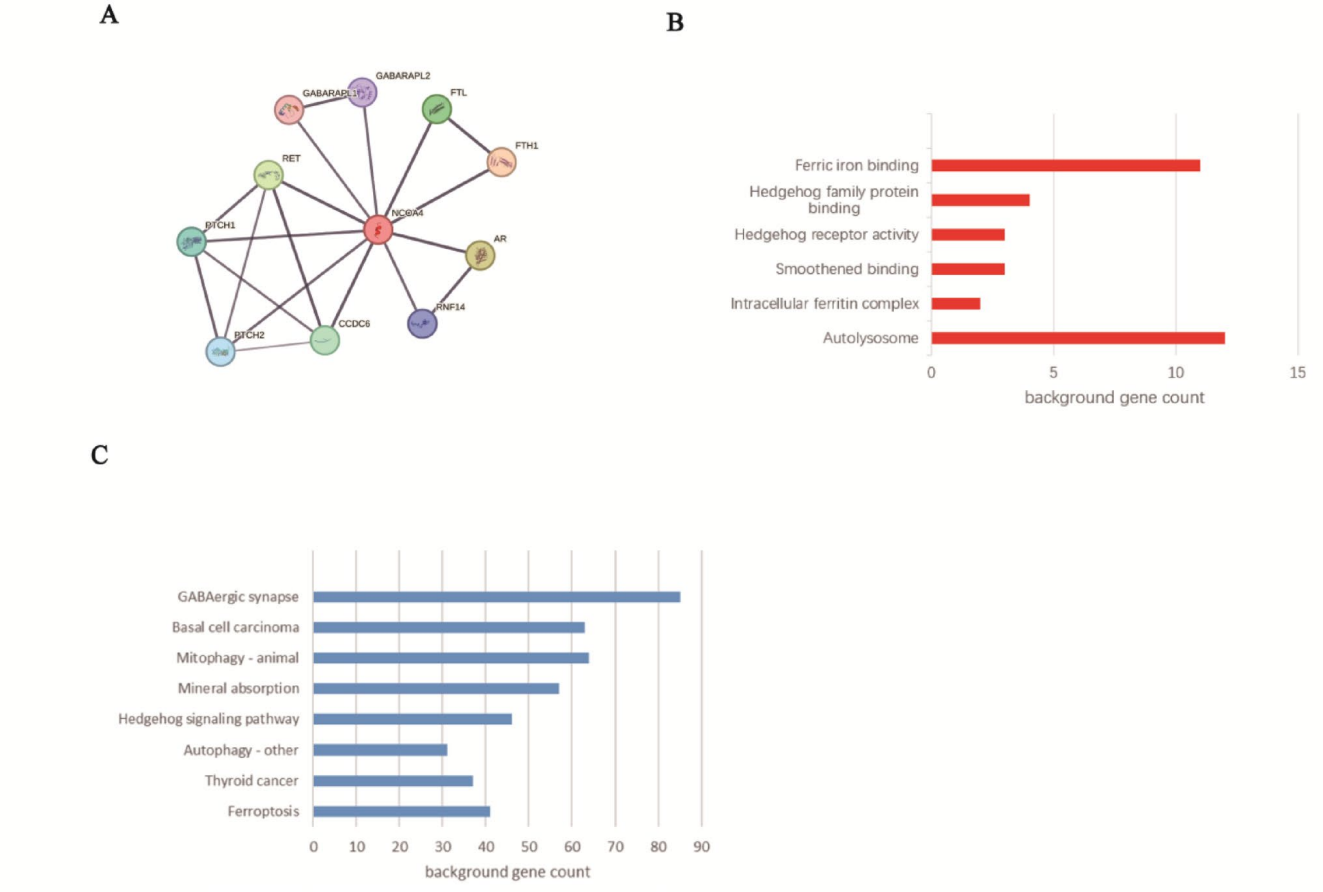
Coexpression network and potential function of NCOA4. **A** Protein–protein interaction (PPI) networks. **B**, **C** GO and KEGG analyses and terms significantly enriched in NCOA4-coexpressed genes in glioma

As shown in Fig. 4A, FTH1, FTL, AR, CCDC6, GABARAPL1, GABARAPL2, PTCH1, PTCH2, RET, and RNF14 were identified as hub genes associated with NCOA4. GO and KEGG analyses were performed on the top-ranked genes coexpressed with NCOA4; the results indicated that these genes were involved mainly in ferroptosis and the Hh signalling pathway (Fig. 4B, C). Because the role of NCOA4 in ferroptosis has been reported in many studies, we focused on PTCH1, a gene that is coexpressed with NCOA4 and whose encoded protein is a major component of the Hh signalling pathway.

### NCOA4 inhibits the aggressive behaviours of glioma cells through the SHH pathway

**Fig. 5:**
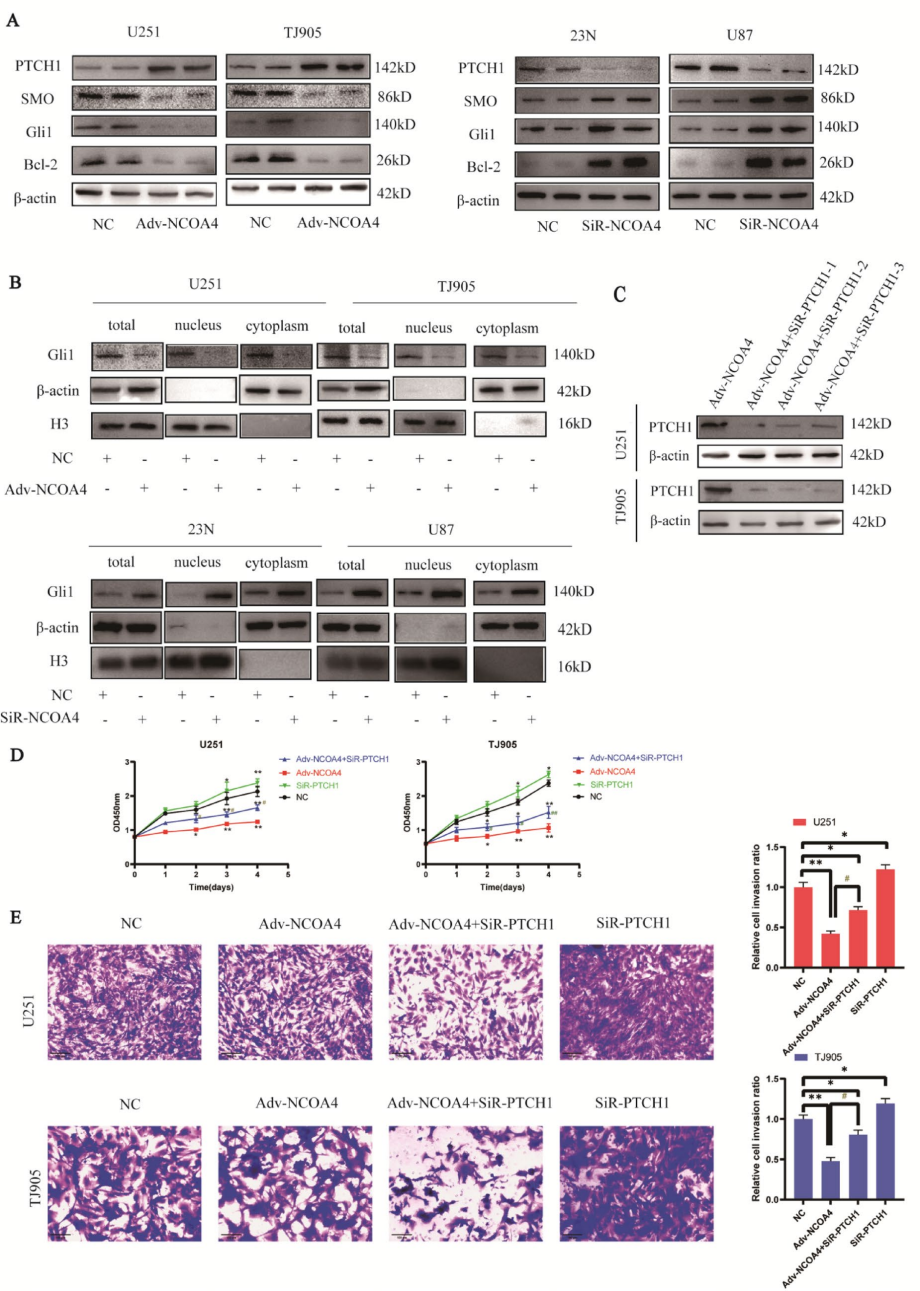
NCOA4 suppresses the growth of glioma cells by inhibiting the SHH pathway. **A** Expression of PTCH1, SMO, Gli1 and Bcl-2 in glioma cell lines transduced with Adv-NCOA4 and siRNA-NCOA4. **B** Total Gli1 expression and its distribution in the cytoplasm and nucleus were assessed by WB. **C** PTCH1 was knocked down in NCOA4-overexpressing cell lines. **D** CCK-8 assay in U251 and TJ905 cell lines. **E** Transwell assays were used to assess the invasion ability of glioma cell lines (× 200). *, *P* < 0.05; **, *P* < 0.01, compared with the control group. #, *P* < 0.05; ##, *P* < 0.01, compared with the Adv-NCOA4 group

We found that PTCH1 was upregulated when NCOA4 was overexpressed in in U251 and TJ905 cells, whereas PTCH1 was downregulated when NCOA4 was knocked down in 23N and U87 cells (Fig. 5A). The effect of NCOA4 on the SHH pathway was further investigated; when NCOA4 was upregulated in U251 and TJ905 cells, SMO was downregulated (Fig. 5A). Total, nuclear and cytoplasmic Gli1 protein expression was downregulated in NCOA4-overexpressing cells compared to control cells. These data revealed that NCOA4 overexpression represses SMO and Gli1, inhibits Gli1 translocation into the cell nucleus and suppresses Gli1 activation. H3 is a specific marker of the nucleus, and β-actin is a specific marker of the cytoplasm (Fig. 5B). Bcl-2, the target gene of Gli1, was found to be downregulated in NCOA4-overexpressing cells (Fig. 5A), which further confirmed the decrease in Gli1 transcriptional activity.^12^ When NCOA4 was knocked down in 23N and U87 cells, SMO and Bcl-2 were upregulated(Fig. 5A). Total, nuclear and cytoplasmic Gli1 protein expression was upregulated in NCOA4 knockdown cells compared to control cells. These data revealed that NCOA4 knockdown promotes SMO and Gli1, also promotes Gli1 translocation into the cell nucleus and promotes Gli1 activation. (Fig. 5B). NCOA4 has been shown to suppress the SHH pathway.

To investigate whether NCOA4 exerts its inhibitory effect on glioma cell growth via the SHH pathway through the regulation of PTCH1, we knocked down PTCH1 in NCOA4-overexpressing GBM cells by transfecting them with siRNA-PTCH1 (Fig. 5C). The proliferative activity of the NCOA4 overexpression combined with PTCH1 knockdown group was significantly greater than that of the NCOA4-overexpression alone group (*p* < 0.05), whereas the proliferative activity of the NCOA4 overexpression combined with PTCH1 knockdown group and the NCOA4-overexpression alone group was significantly lower than that of the control group, the proliferative activity of the PTCH1 knockdown alone group was greater than the control group (*P* < 0.05) (Fig. 5D). These results demonstrated that PTCH1 knockdown partly reversed the inhibitory effect of NCOA4 on GBM cell proliferation. Compared with that in the NCOA4 overexpression group, the number of invading cells in the NCOA4 overexpression combined with PTCH1 knockdown group was significantly greater (*p* < 0.05), whereas the number of invading cells in the NCOA4 overexpression combined with PTCH1 knockdown group and NCOA4 overexpression alone group was significantly lower than that in the control group, the number of invading cells in the PTCH1 knockdown alone group was greater than the control group (*P* < 0.05) (Fig. 5E), which suggested that downregulation of PTCH1 partially reversed the suppressive effect of NCOA4 on tumour cell invasion.

These findings indicate that NCOA4 might suppress glioma progression partially through the SHH pathway.

## Discussion

After decades of development, great advances have been made in glioma treatment. A better understanding of the pathogenesis of gliomas may reveal novel therapeutic targets and innovative treatment modalities. NCOA4 has been reported to play a tumour-suppressive role in prostate and breast cancers (Anestis et al. 2020; Aurilio et al. 2020), but its role in gliomas remains unclear. Chen et al. reported that NCOA4 was upregulated in gliomas compared with nontumorous brain tissues but decreased in glioma cells (Chen et al. 2024). In the present study, TCGA data analysis revealed that NCOA4 is downregulated in a variety of tumours including gliomas. DNA methylation is a major epigenetic modification related to gene expression that has been extensively researched in various cancers. Many studies have focused on the association between the expression and methylation of specific genes in tumours, but the relationship between NCOA4 and its methylation has not been studied in gliomas (Zhang et al. 2019; Wang et al. 2018). In this study, a negative correlation between NCOA4 expression and methylation was observed in glioma samples. NCOA4 hypomethylation and high expression also predicted better OS in glioma patients. The expression of NCOA4 was further detected in glioma cells and specimens by WB and IHC. NCOA4 expression was downregulated in glioma cell lines and specimens compared with nontumorous brain tissues and was negatively correlated with glioma grade. Patients with high NCOA4 expresison had longer survival than those with low expression.

To determine the effect of NCOA4 on glioma cell growth, NCOA4 was overexpressed in glioma cells; NCOA4 overexpression significantly inhibited GBM cell growth and invasion and induced apoptosis. While the proliferation and invasion of glioma cells were promoted, apoptosis was inhibited when NCOA4 was knocked down. Biomarkers related to proliferation, invasion and apoptosis, such as PCNA, MMP2 and caspase-3, were downregulated when NCOA4 was overexpressed. These results demonstrated that NCOA4 might play an inhibitory role in glioma progression.

Previous studies have suggested that NCOA4 can increase intracellular iron levels and subsequently promote ferroptosis through the degradation of ferritin to regulate glioma progression (Lin et al. 2023; Li et al. 2022). In our study, bioinformatics analysis revealed an interaction between NCOA4 and PTCH1. PTCH1 is a suppressor of the SHH pathway. The SHH pathway plays an important role in central nervous system (CNS) tumours. TRIM37 knockdown can promote glioma cell apoptosis through the inhibition of the SHH signalling pathway by increasing the expression of PTCH1 (Cai et al. 2024). HDAC6 suppresses glioma cell proliferation and promotes glioma cell apoptosis via inactivation of the SHH/Gli1 signalling pathway (Yang et al. 2018). In addition, a powerful negative regulator of the HH signalling pathway (SUFU) suppresses glioma cell growth, invasion and angiogenesis by affecting the subcellular distribution of Gli1 (Liu et al. 2014). Activation of the Hedgehog signalling pathway can also promote the growth of oligodendrogliomas and astrocytomas (Zhu et al. 2011; Mao et al. 2009). To our knowledge, the relationship between NCOA4 and the SHH pathway has not been reported.

As shown by our results, Overexpression of NCOA4 upregulates PTCH1 expression, NCOA4 knockdown downregulates PTCH1 expression. Overexpression of NCOA4 led to further inhibition of SMO expression and then suppression of Gli1 expression and nuclear translocation; this inhibits the expression of Bcl-2, the target gene of the SHH pathway, leading to the suppression of the SHH pathway. Knockdown of PTCH1 partially reversed the inhibitory effects of NCOA4 on GBM cell growth and invasion, suggesting that PTCH1, a potential downstream gene of NCOA4, is involved in the suppressive effect of NCOA4 on glioma development. In this study, NCOA4 was shown to repress glioma progression partially through inhibiting the SHH pathway. We discovered for the first time that NCOA4 can regulate glioma progression through the SHH pathway, not just by mediating ferroptosis.

## Conclusion

In summary, bioinformatics analysis and our experiments revealed that NCOA4 expression is downregulated in gliomas and is negatively correlated with glioma grade. Moreover, glioma patients with higher NCOA4 expression exhibit longer OS, and NCOA4 suppresses glioma progression through the SHH pathway. These results suggest that NCOA4 might be a promising target for glioma treatment.

## Data Availability

The data generated in the present study may be requested from the corresponding author.

## References

[CR1] Anestis A, Zoi I, Papavassiliou AG, Karamouzis MV (2020) Androgen receptor in breast cancer-clinical and preclinical research insights. Molecules 25(2):358. 10.3390/molecules25020358 31952272 10.3390/molecules25020358 PMC7024330

[CR2] Aurilio G, Cimadamore A, Mazzucchelli R, Lopez-Beltran A, Verri E, Scarpelli M et al (2020) Androgen receptor signaling pathway in prostate cancer: from genetics to clinical applications. Cells 9(12):2653. 10.3390/cells9122653 33321757 10.3390/cells9122653 PMC7763510

[CR3] Cai L, Liu Y, Li Y, Liu B, Cao YuFei, Yang W et al (2024) TRIM37 interacts with EZH2 to epigenetically suppress PTCH1 and regulate stemness in glioma stem cells through sonic hedgehog pathway. J Neurooncol 169(2):269–279. 10.1007/s11060-024-04726-y 38884661 10.1007/s11060-024-04726-y

[CR4] Chen G, Shi X, Zeng Xi, Jiao R (2024) Opposite expression of NCOA4 in glioblastoma tissues and cell lines. Int Immunopharmacol 143(Pt 1):113356. 10.1016/j.intimp.2024.113356 39383786 10.1016/j.intimp.2024.113356

[CR5] Dolecek TA, Propp JM, Stroup NE, Kruchko C (2012) CBTRUS statistical report: primary brain and central nervous system tumors diagnosed in the United States in 2005–2009. Neuro Oncol 14(Suppl 5):1–49. 10.1093/neuonc/nos218 10.1093/neuonc/nos218 PMC3480240 23095881

[CR6] Du W, Liu X, Chen L, Dou Z, Lei X, Chang L et al (2015) Targeting the SMO oncogene by miR-326 inhibits glioma biological behaviors and stemness. Neuro Oncol 17(2):243–253. 10.1093/neuonc/nou217 25173582 10.1093/neuonc/nou217 PMC4288524

[CR7] Hao Y, Li Z, Zhang A, Sun L, Wang G, Wang H et al (2023) The role of PKN1 in glioma pathogenesis and the antiglioma effect of raloxifene targeting PKN1. J Cell Mol Med 27(18):2730–2743. 10.1111/jcmm.17860 37480215 10.1111/jcmm.17860 PMC10494285

[CR8] Hou W, Xie Y, Song X, Sun X, Lotze MT, Zeh HJ et al (2016) Autophagy promotes ferroptosis by degradation of ferritin. Autophagy 12(8):1425–1428. 10.1080/15548627.2016.1187366 27245739 10.1080/15548627.2016.1187366 PMC4968231

[CR9] Kollara A, Brown TJ (2012) Expression and function of nuclear receptor co-activator 4: evidence of a potential role independent of co-activator activity. Cell Mol Life Sci 69(23):3895–3909. 10.1007/s00018-012-1000-y 22562579 10.1007/s00018-012-1000-y PMC3492700

[CR10] Li J, Cai J, Zhao S, Yao K, Sun Y, Li Y et al (2016) GANT61, a GLI inhibitor, sensitizes glioma cells to the temozolomide treatment. J Exp Clin Cancer Res 35(1):184. 10.1186/s13046-016-0463-3 27894350 10.1186/s13046-016-0463-3 PMC5127098

[CR11] Li K, Chen B, Aibo Xu, Shen J, Li K, Hao Ke et al (2022) TRIM7 modulates NCOA4-mediated ferritinophagy and ferroptosis in glioblastoma cells. Redox Biol 56:102451. 10.1016/j.redox.2022.102451 36067704 10.1016/j.redox.2022.102451 PMC9468590

[CR12] Ligr M, Li Y, Zou X, Daniels G, Melamed J, Peng Yi et al (2010) Tumor suppressor function of androgen receptor coactivator ARA70alpha in prostate cancer. Am J Pathol 176(4):1891–1900. 10.2353/ajpath.2010.090293 20167864 10.2353/ajpath.2010.090293 PMC2843478

[CR13] Lin Y, Gong H, Liu J, Zhiwen Hu, Gao M, Wei Yu et al (2023) HECW1 induces NCOA4-regulated ferroptosis in glioma through the ubiquitination and degradation of ZNF350. Cell Death Dis 14(12):794. 10.1038/s41419-023-06322-w 38049396 10.1038/s41419-023-06322-w PMC10695927

[CR14] Liu X, Wang X, Wenzhong Du, Chen L, Wang G, Cui Y et al (2014) Suppressor offused (Sufu) represses Gli1 transcription and nuclear accumulation, inhibits glioma cell proliferation, invasion and vasculogenic mimicry, improving glioma chemo-sensitivity and prognosis. Oncotarget 5(22):11681–11694. 10.18632/oncotarget.2585 25373737 10.18632/oncotarget.2585 PMC4294353

[CR15] Loui DN, Perry A, Wesseling P, Brat DJ, Cree IA, Figarella-Branger D et al (2021) The 2021 WHO classification of tumors of the central nervous system: a summary. Neuro Onco 23(8):1231–1251. 10.1093/neuonc/noab106 10.1093/neuonc/noab106 PMC8328013 34185076

[CR16] Mao L, Xia YP, Zhou YN, Dai R-L, Yang X, Duan S-J et al (2009) A critical role of Sonic hedgehog signaling in maintaining the tumorigenicity of neuroblastoma cells. Cancer Sci 100(10):1848–1855. 10.1111/j.1349-7006.2009.01262.x 19622100 10.1111/j.1349-7006.2009.01262.x PMC11158460

[CR17] Mestayer C, Blanchère M, Jaubert F, Dufour B, Mowszowicz I (2003) Expression of androgen receptor coactivators in normal and cancer prostate tissues and cultured cell lines. Prostate 56(3):192–200. 10.1002/pros.10229 12772188 10.1002/pros.10229

[CR18] Santana-Codina N, Gikandi A, Mancias JD (2021) The Role of NCOA4-Mediated Ferritinophagy in Ferroptosis. Adv Exp Med Biol 1301:41–57 34370287 10.1007/978-3-030-62026-4_4

[CR19] Sigafoos AN, Paradise BD, Fernandez-Zapico ME (2021) Hedgehog/GLI signaling pathway: transduction, regulation, and implications for disease. Cancers (Basel) 13(14):3410. 10.3390/cancers13143410 34298625 10.3390/cancers13143410 PMC8304605

[CR20] Wang Z, Wang Z, Zhang C, Liu X, Li G, Liu S et al (2018) Genetic and clinical characterization of B7–H3 (CD276) expression and epigenetic regulation in diffuse brain glioma. Cancer Sci 109(9):2697–2705. 10.1111/cas.13744 30027617 10.1111/cas.13744 PMC6125452

[CR21] Wang LM, Englander ZK, Miller ML, Bruce JN, Glioma M (2023) human brain and spinal cord tumors: from bench to bedside. Adv Exp Med Biol 1405:1–30. 10.1007/978-3-031-23705-8_1 37452933 10.1007/978-3-031-23705-8_1

[CR22] Wen-Zhong Du, Feng Y, Wang X-F, Piao X-Y, Cui Y-Q, Chen L-C et al (2013) Curcumin suppresses malignant glioma cells growth and induces apoptosis by inhibition of SHH/GLI1 signaling pathway in vitro and vivo. CNS Neurosci Ther 19(12):926–936. 10.1111/cns.12163 24165291 10.1111/cns.12163 PMC6493544

[CR23] Xinyu Wu, Chen F, Sahin A, Albarracin C, Pei Z, Zou X et al (2011) Distinct function of androgen receptor coactivator ARA70alpha and ARA70beta in mammary gland development,and in breast cancer. Breast Cancer Res Treat 128(2):391–400. 10.1007/s10549-010-1131-5 20814820 10.1007/s10549-010-1131-5

[CR24] Yang W, Liu Y, Gao R, Hongquan Yu, Sun T (2018) HDAC6 inhibition induces glioma stem cells differentiation and enhances cellular radiation sensitivity through the SHH/Gli1 signaling pathway. Cancer Lett 28(415):164–176. 10.1016/j.canlet.2017.12.005 10.1016/j.canlet.2017.12.005 29222038

[CR25] Zhang H, Zhang L, Tang Y, Wang C, Chen Y, Shu J et al (2019) Systemic screening identifies GABRD, a subunit gene of GABAA receptor as a prognostic marker in adult IDH wild-type diffuse low-grade glioma. Biomed Pharmacother 118:109215. 10.1016/j.biopha.2019.109215 31545245 10.1016/j.biopha.2019.109215

[CR26] Zhu W, You Z, Li T, Changhua Yu, Tao G, Mingli Hu et al (2011) Correlation of hedgehog signal activation with chemoradiotherapy sensitivity and survival in esophageal squamous cell carcinomas. Jpn J Clin Oncol 41(3):386–393. 10.1093/jjco/hyq217 21127038 10.1093/jjco/hyq217

